# Phospholipid binding to the FAK catalytic domain impacts function

**DOI:** 10.1371/journal.pone.0172136

**Published:** 2017-02-21

**Authors:** Jessica E. Hall, Michael D. Schaller

**Affiliations:** 1 Department of Biochemistry, West Virginia University School of Medicine, Morgantown, West Virginia, United States of America; 2 Mary Babb Randolph Cancer Center, West Virginia University School of Medicine, Morgantown, West Virginia, United States of America; Oregon State University, UNITED STATES

## Abstract

Focal adhesion kinase is an essential nonreceptor tyrosine kinase that plays an important role in development, in homeostasis and in the progression of human disease. Multiple stimuli activate FAK, which requires a change in structure from an autoinhibited to activated conformation. In the autoinhibited conformation the FERM domain associates with the catalytic domain of FAK and PI(4,5)P_2_ binding to the FERM domain plays a role in the release of autoinhibition, activating the enzyme. An in silico model of FAK/PI(4,5)P_2_ interaction suggests that residues on the catalytic domain interact with PI(4,5)P_2_, in addition to the known FERM domain PI(4,5)P_2_ binding site. This study was undertaken to test the significance of this in silico observation. Mutations designed to disrupt the putative PI(4,5)P_2_ binding site were engineered into FAK. These mutants exhibited defects in phosphorylation and failed to completely rescue the phenotype associated with *fak* ^*-/-*^ phenotype fibroblasts demonstrating the importance of these residues in FAK function. The catalytic domain of FAK exhibited PI(4,5)P_2_ binding in vitro and binding activity was lost upon mutation of putative PI(4,5)P_2_ binding site basic residues. However, binding was not selective for PI(4,5)P_2_, and the catalytic domain bound to several phosphatidylinositol phosphorylation variants. The mutant exhibiting the most severe biological defect was defective for phosphatidylinositol phosphate binding, supporting the model that catalytic domain phospholipid binding is important for biochemical and biological function.

## Introduction

Focal adhesion kinase (FAK) is an essential non-receptor tyrosine kinase since *fak*^-/-^ mice exhibit embryonic lethality. FAK is broadly expressed in different cell types and loss of FAK expression or function results in multiple embryonic defects including deficits in angiogenesis, formation of the neural tube and development of a multi-chambered heart [1–3]. Homeostasis in some adult tissues is also dependent upon FAK, as demonstrated in conditional FAK knockout mouse models. For example, in keratinocytes FAK is necessary for maintenance of an epidermis of normal thickness and for sebaceous gland function, and in intestinal epithelial cells, FAK is required for efficient mucosal wound healing [4,5]. FAK also plays a role in the development of pathologies associated with several human diseases. The most extensive evidence has implicated FAK in the development and progression of a number of cancers and significant efforts to therapeutically target FAK with small ATP analog inhibitors have been made [6,7]. FAK has also been implicated in atherosclerosis, and the tissue remodeling that occurs during cardiac hypertrophy [8–10]. At the cellular level, FAK regulates cell proliferation, cell survival, and cell migration, and control of these cellular events underpins the biological functions of FAK in development, homeostasis, and disease [11–13].

Given its broad distribution of expression across tissues and role in controlling multiple cellular and biological functions, it is not surprising that a wide range of stimuli can activate FAK signaling. Integrin-dependent cell adhesion to extracellular matrix proteins is a major activating signal and soluble ligands for receptor tyrosine kinases and G protein coupled receptors can trigger FAK activation [14]. These diverse stimuli initiate catalytic activation of FAK and autophosphorylation, which creates a binding site for Src, another nonreceptor tyrosine kinase, which in turn phosphorylates FAK at additional sites [15–17]. Tyrosine phosphorylation of FAK stimulates maximal catalytic activity in addition to regulating FAK’s ability to serve as a scaffold to assemble a complex of signaling molecules to transduce downstream signals [18].

**Fig 1:**
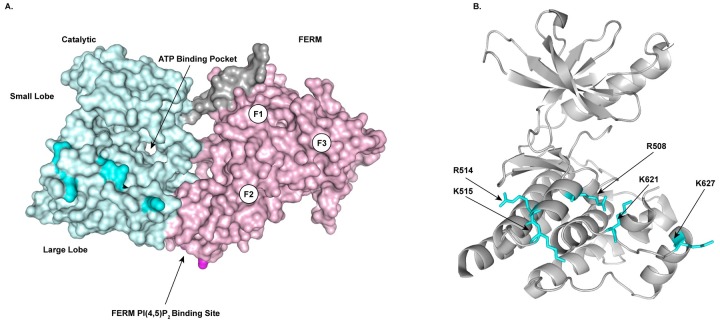
Predicted PI(4,5)P_2_ binding sites on the FAK catalytic domain. **A)** The surface view of the FERM (pink) and catalytic (blue) domains in the autoinhibited conformation is shown. The linker connecting the FERM and catalytic domains is colored gray. The F1, F2 and F3 lobes of the FERM domain and the position of the PI(4,5)P_2_ binding site are indicated. The basic residues in the large lobe of the catalytic domain that are proposed to bind PI(4,5)P_2_ are colored cyan. **B)** Ribbon diagram of the catalytic domain of FAK demonstrating the location of the catalytic domain residues proposed to bind PI(4,5)P_2_.

The critical event in FAK activation is a conformation change from an autoinhibited to an activated state. In the autoinhibited conformation, the FAK band 4.1, Ezrin, Radixin, Moesin (FERM) domain binds to the catalytic domain to occlude the active site and substrate binding site [19]. There are two contact sites in this conformation, one between the C-terminal lobe of the kinase domain and the F2 lobe of the FERM domain and the second between the N-terminal lobe of the kinase domain, the linker between the FERM and kinase domains, and the F1 lobe of the FERM domain (Fig 1). Activating stimuli are proposed to modify the FERM domain or generate a FERM domain binding ligand that reduces the affinity of the FERM domain for the catalytic domain relieving the inhibitory interaction and/or stabilizing the active conformation. Phosphorylation of FAK at tyrosine 194 by the Met receptor tyrosine kinase is proposed to relieve autoinhibition in response to hepatocyte growth factor stimulation, and Src-dependent phosphorylation of the activation loop of FAK is believed to sterically block FERM domain binding to the catalytic domain, thus stabilizing the active conformation [19,20]. A change in intracellular pH also modulates FAK activity through a mechanism of protonation/deprotonation of histidine 75, which resides in the FERM domain and is proposed to alter the stability of the autoinhibited conformation [21]. A FERM domain basic sequence in the F2 lobe lies near the interface with the kinase domain and is required for FAK activation [22]. This sequence serves as a binding site for two different ligands, a tyrosine phosphorylated peptide from the Met receptor and PI(4,5)P_2_ [23,24]. Binding of these ligands to the basic sequence in the FERM domain is envisioned to relieve autoinhibition.

The role of PI(4,5)P_2_ binding in FAK activation has been experimentally addressed using biochemical and computational approaches. FAK binding to PI(4,5)P_2_ is cooperative and induces FAK clustering *in vitro* [25]. The FERM domain mediates FAK dimerization, which is required for biochemical and biological function *in vivo* [26]. However, dimerization occurs *in vitro* in the absence of PI(4,5)P_2_ and thus the role of this interaction in PI(4,5)P_2_-induced clustering has not been elucidated. Molecular dynamics simulations have been performed to gain insight into the molecular mechanism of FAK activation by PI(4,5)P_2_. Allosteric connectivity is observed between alpha helix C and alpha helix G, which reside in the N-terminal and C-terminal lobes of the catalytic domain respectively. Alpha helix C contacts the linker, which contacts the F1 lobe of the FERM domain, and alpha helix G directly contacts the F2 lobe of the FERM domain in the autoinhibited state [19,25]. PI(4,5)P_2_ docking induces local changes in the F2 lobe of the FERM domain, and also induces long range changes between the N-terminal lobe of the kinase domain and the F1 lobe of the FERM domain [25,27]. In these simulations, PI(4,5)P_2_ binding induced small changes in the conformation of the FERM/kinase domain and did not cause dissociation of the two domains [25,27,28]. Additional events may therefore contribute to the conversion of the autoinhibited to fully activated state. Molecular dynamics simulations have demonstrated that PI(4,5)P_2_ sufficiently tethers the FERM domain to allow mechanical separation of the FERM and catalytic domains when forces are applied to the C-terminus of the catalytic domain [29]. This is an interesting model given FAK’s role in sensing mechanical stiffness of the extracellular matrix and the requirement of an intact actin cytoskeleton for activation [30–32]. Interestingly, one of the molecular dynamics simulation studies suggested a novel interaction between the catalytic domain and PI(4,5)P_2_ [28]. In addition to the interaction of the F2 lobe basic sequence with PI(4,5)P_2_, basic residues in the catalytic domain also docked with PI(4,5)P_2_ in these simulations. Of interest were a number of basic residues on the side of the large lobe of the catalytic domain as a rotation of the catalytic domain with respect to the FERM domain might be required for membrane binding, and this movement could potentially contribute mechanistically to FAK activation. The current study was undertaken to test the biochemical and biological significance of this observation.

## Materials and methods

### Molecular biology

FAK mutants were created using a modified version of the Quickchange (Agilent) mutagenesis protocol using the viral vector pLUdr containing wild type avian FAK cDNA or the expression vector pGEX-KG containing wild type avian FAK catalytic domain as a template. Primers for site-directed mutagenesis were designed to substitute alanine for lysine or arginine residues at the sites of interest. Sequence analysis verified the presence of the intended mutations and that no unintended mutations were present. (ACGT Inc.).

### Cell culture and imaging

Human embryonic kidney (HEK) 293T cells and mouse embryonic fibroblasts (MEFs) (ATCC) were maintained in Dulbecco’s modified Eagle’s medium (DMEM) containing 10% fetal bovine serum (FBS) and 1% Streptomycin/Penicillin. HEK 293T cells were co-transfected with the pLUdr-FAK vectors and the PAX2 and VSVG viral packaging vectors using Turbofect (Thermo Fisher) according to the manufacturer’s instructions. Media containing virus was collected at 24, 48, and 72 hours. Media was pooled and filtered using a 0.45 μm filter (Milipore). *fak*^-/-^ MEFs (ATCC) were incubated with the resulting virus-containing supernatant for 6 hours and selected with puromycin after 24 hours. Puromycin selection was carried out for five days.

For immunofluorescence experiments, cells were plated on fibronectin coated coverglass overnight. Cells were fixed in 3% formaldehyde for 15 min and permeabilized with 0.4% Triton X-100 for 10 min. FAK was detected using the BC4 anti-FAK antibody and an Alexa Flour488 (Invitrogen) conjugated anti-rabbit secondary antibody as previously described [22]. Paxillin was detected using an anti-paxillin antibody (BD Biosciences) and an Alexa Fluor 488 conjugated anti-mouse secondary antibody (Invitrogen). Cells were visualized by using a Zeiss Fluorescent Axio Imager Z2 microscope with Zeiss 63x (N.A. 1.40) objective. Images were taken with an AxioCam MRm Rev.3 camera with identical exposure times. Images were analyzed using ImageJ. Individual cells were selected and the contrast inverted. The threshold of this overlay was set to restrict analysis to focal adhesions. The area of focal adhesions was calculated using the Analyze Particles function. The scale was set so that pixel area was converted into μM^2^.

To measure cell spreading, *fak*^-/-^ cells expressing wild type or mutant FAK were transfected with GFP-paxillin to allow live cell imaging. Cells were plated on fibronectin coated imaging glass dishes. After a five minute incubation period to allow attachment, dishes were placed in an environmental chamber mounted onto a Nikon Swept Field confocal microscope. Cells expressing GFP-paxillin were visualized using a Nikon 40x oil objective and images were taken at one minute intervals with an exposure time of 200 msec for GFP. Background subtraction was performed. Cells were outlined and cell diameters at each time point were recorded using the Nikon NIS Elements analysis software.

To measure focal adhesion dynamics *fak*^*-/-*^ cells expressing wild type or mutant FAK were transfected with GFP-paxillin to allow identification of adhesions. Cells were placed on fibronectin coated imaging glass dishes for 4 hours to allow for spreading defects between cell lines. Cells were refed with imaging media and placed in an environmental chamber mounted on a Nikon Swept Field confocal microscope. Cells were visualized utilizing a Nikon 60x oil objective and images were taken at one minute intervals with an exposure time of 200 msec over an hour. Background subtraction was performed. Focal adhesions were analyzed utilizing the Focal Adhesion Analysis Server [33,34]. This server allows the log-linear fitting method determination of assembly and disassembly phase length in which models are fit to all assembly and disassembly phases greater than or equal to a length of 2 minutes, as described in Webb et al. [35].

### Protein analysis

Cells were lysed in ice-cold modified radioimmunoprecipitation assay (RIPA) buffer (50 mM Tris-HCl [pH 7.3], 150 mM NaCl, 1% IGEPAL, 1% Nonidet P-40, 0.5% deoxycholate, 0.5% aprotinin, 1 mM phenylmethylsulfonyl fluoride, 1.5 mM vanadate). Lysates were clarified, and protein concentrations were determined using the bicinchoninic acid assay (Pierce). Lysates were boiled in Laemmli sample buffer (60 mM Tris-Cl pH 6.8, 2% S.D.S, 10% glycerol, 5% β-mercaptoethanol, 0.01% bromophenol blue) and 15 μg of total protein was analyzed by Western blotting. An anti-FAK antibody (4.47—Millipore), an anti-phosphotyrosine antibody (4G10—Millipore) and anti-FAK phosphospecific antibodies (PTyr397, PTyr577 and PTyr861—Invitrogen) were used as primary antibodies for Western blotting. Horseradish peroxidase (HRP) conjugated anti-mouse and anti-rabbit secondary antibodies (Millipore) were used to detect FAK expression and phosphorylation levels using Immobilon Western HRP chemiluminescence substrate (Millipore). Quantification of blots was performed with ImageJ (http://rsb.info.nih.gov/nih-image). Developed films were scanned as TIFF images in 8-bit grayscale format at 600 dpi. The lanes were defined using the rectangular select tool and the Analyze → Gels → Select First Lane/Select Next Lane function. Densitometry measurements were calculated using Analyze → Gels → Plot lanes. Band peak was defined from background using the straight line tool then the area of the band peak was calculated using the Wand tool. (Protocol can be found at http://rsb.info.nih.gov/nih-image/manual/tech.html#analyze.) Band intensities of the phosphotyrosine blots were divided by the band intensities of the FAK blots and all wild type samples were normalized to one.

### Protein purification

The expression of recombinant protein in Escherichia coli BL21 (Codon Plus) cells (Agilent) was induced at an absorbance (OD) at 600 nm of 0.8 to 1.0 by the addition of 0.1 mM IPTG (isopropyl-β-d-thiogalactopyranoside), and cells were grown for an additional 8 h at 22°C. The cells were harvested and frozen at −20°C. Cell pellets were thawed and resuspended in lysis buffer (20 mM Tris [pH 8.0], 100 mM NaCl, 1 mM ethylenediaminetetraacetic acid, 4% Triton X-100, 10% sarkosyl, 10 mM 3-[(3-cholamidopropyl)dimethylammonio]-1-propanesulfonate (CHAPS)). Lysis buffer was supplemented with the addition of protease inhibitors at a final concentration of 1 mM phenylmethylsulfonyl fluoride, 1 mM benzamadine, 10 μg/ml of leupeptin, 10 mg/ml of lysozyme, and 1 mg/ml of DNase. The resuspended cells were lysed by sonication on ice. After clarification, supernatants were loaded onto a glutathione sepharose column. Following extensive washing with Tris buffered saline (pH 7.4), protein was eluted with a buffer containing 50 mM Tris [pH 8.0], 150 mM NaCl and 50 mM glutathione. Elution fractions were concentrated using Ultra-4 centrifugal filter units (10 kDa NMWL) (Amicon).

### Lipid binding

Lipid binding was assessed by co-sedimentation with large, unilamellar vesicles (LUV). The ability of the catalytic domain to bind PI(4,5)P_2_ was analyzed using lipid vesicles containing 50%PC/40%PE/10% PI(4,5)P_2_ (experimental) or 60%PC/40%PE (control). Vesicles were prepared by mixing chloroform dissolved phospholipids (Echelon) in appropriate ratios. The mixture was dried using a speed vacuum for 15 minutes. The dried lipid cake was suspended into a lipid binding buffer (20 mM HEPES [pH 7.5], 2 mM dithiothreitol, 250 mM NaCl) to a final concentration of 2.5 μg/ul [36]. The lipid suspension containing large multilamellar vesicles (LMV) was passed eleven times through a 100 nm filter using an Avanti mini-extruder set to ensure a homogenous suspension of LUVs. Four micrograms of glutathione S-transferase (GST) fusion protein was incubated with 250 μg of lipid vesicles on ice for 1h. The mixtures were centrifuged at 100,000 x g for 1h at 4 C°. The supernatants were collected, mixed with Laemmli sample buffer (60 mM Tris-Cl pH 6.8, 2% S.D.S, 10% glycerol, 5% β-mercaptoethanol, 0.01% bromophenol blue) and boiled. The pellets were resuspended in Laemmli sample buffer and boiled. The samples were analyzed by S.D.S-polyacrylamide gel electrophoresis (PAGE) and Coomassie blue staining. Quantification of gels was performed with ImageJ (http://rsb.info.nih.gov/nih-image). Images of gels were converted to TIFF images in 8-bit grayscale format at 600 dpi. The lanes were defined using the rectangular select tool and the Analyze → Gels → Select First Lane/Select Next Lane function. Densitometry measurements were calculated using Analyze → Gels → Plot lanes. Band peak was defined from background using the straight line tool, then the area of the band peak was calculated using the Wand tool. (Protocol can be found at http://rsb.info.nih.gov/nih-image/manual/tech.html#analyze.) Band intensities of the supernatant and pellet lanes were divided by the sum of the band intensities of the supernatant and pellet.

For anisotropy experiments purified GST fusion proteins were incubated with BODIPY (dipyrromethene boron difluoride)-labeled phosphoinosotides with C6-acyl chains (Echelon). Increasing concentrations of purified protein were added to 12.5 nM fluorescent phosphoinositide in buffer containing 20 mM HEPES [pH7.5], 150 mM NaCl, and 5 mM β-mercaptoethanol. Anisotropy measurements were taken at 37°C using a Flourlog spectroflourometer equipped with Glan-Thompson polarizers with FluorEssence software (HORIBA Jobin Yvon). Specific binding of the catalytic domain was determined by subtraction of binding of GST alone. Binding curves and dissociation constants were determined using the Prism 5.0 statistical analysis software (GraphPad).

### Kinase assay

White low-volume 96-well polystyrene plates (PerkinElmer) were used for the ADP-Glo assay. Four μg of kinase was mixed with 25 μL of kinase buffer (5 mM MgCl2, 5 mM MnCl, 50 mM TrisHCl pH 7.4), 2.5 μg Poly-Glu-Tyr, and 25 mM ultrapure ATP (Promega). All reactions were carried out in triplicate. Blank wells lacked enzyme but did include kinase buffer, substrate, and ATP. The plates were covered and the reactions were carried out at room temperature (RT) for up to 120 min. Reactions were stopped with the addition of 50 μL ADP-Glo reagent (Promega). After a 40-min incubation at RT, 50 μL of Kinase Detection Reagent (Promega) was added and the plates were incubated for another 40 min at RT. Plates were read on a BioTek Synergy 4 plate reader with a sensitivity of 150 and an integration time of 1 s per well. For kinase reactions involving lipids, 8 μg of kinase was preincubated with 25 μL of lipid vesicle solution at RT prior to addition of 25 μL of kinase buffer with 5 μg poly-Glu-Tyr, 25 mM ATP. Data were plotted using Prism 5.0 statistical analysis software (GraphPad).

## Results

### Catalytic domain residues are required for maximal FAK phosphorylation in vivo

**Fig 2:**
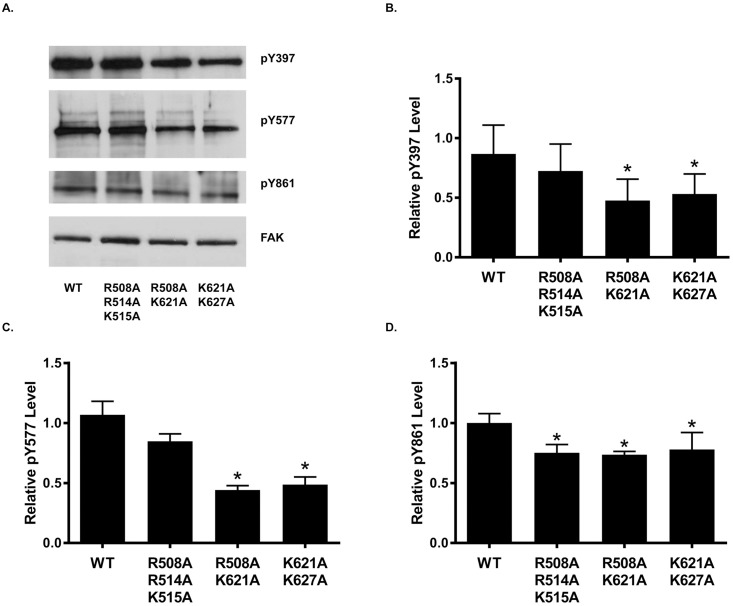
Catalytic domain mutants of FAK exhibit defects in phosphorylation. *fak*^-/-^ MEFs expressing wild type or mutant FAK proteins were analyzed to assess FAK tyrosine phosphorylation. **A**) Representative Western blots of cell lysates probed with the FAK 4.47 antibody (FAK) or the phospho-specific pTyr397, pTyr577, and pTyr861 antibodies are shown. **B-D**) Western blot quantification was performed using ImageJ. Phosphorylation levels were normalized to FAK expression levels. Mutant phosphorylation levels were compared to WT phosphorylation levels using a one-way ANOVA analysis with a Dunnett’s post-test (*n* = 6 [pTyr397] or 10 [pTyr577 and pTyr581] experiments, * = *P* < 0.05).

To assess the physiological relevance of the catalytic domain basic residues implicated in phospholipid binding by the molecular modeling simulations, select residues were substituted with alanine and engineered in the full length FAK cDNA in the pLUdr lentiviral vector (Fig 1). Since Arg508, Arg514 and Lys515 are located in a single alpha helix (αD), a mutant containing alanine substitutions for these residues was engineered (R508A/R514A/K515A). As Lys621 and Lys627 are located within the same loop between αF and αG of the FAK catalytic domain, another mutant with alanines substituted for these two residues was created (K621A/K627A). Finally, since Arg508 and Lys621 are in close proximity in the three-dimensional structure of the catalytic domain, a mutant with alanine replacing these two residues was created (R508A/K621A) (Fig 1B). The R508A/R514A/K515A, R508A/K621A, and K621A/K627A mutants were expressed in *fak*^*-/-*^ mouse embryo fibroblasts (MEFs). Populations of MEFs expressing each construct were lysed and lysates were analyzed by Western blotting for FAK expression and FAK phosphorylation (Fig 2A). Tyrosine phosphorylation levels were normalized to FAK expression levels and compared to WT FAK phosphorylation levels. FAK phosphorylation at Tyr397 and Tyr577 was significantly decreased in the R508A/K621A and K621A/K672A mutants (Fig 2B and 2C). The R508A/R514A/K515A mutant exhibited a reproducible reduction in tyrosine phosphorylation at these sites, but the decrease did not reach statistical significance. All three mutants showed a reproducible reduction in phosphorylation at the Tyr861 site, with the R508A/K621A and R508A/K514A/K515A mutants showing a significant decrease (Fig 2D). Autophosphorylation at Tyr397 is one of the most well defined steps in FAK activation and is necessary for Src binding and subsequent phosphorylation of Tyr577 and Tyr861. The reduction in phosphorylation of these sites is indicative of reduced FAK activation and demonstrates that these mutants are less responsive to upstream signals.

**Fig 3:**
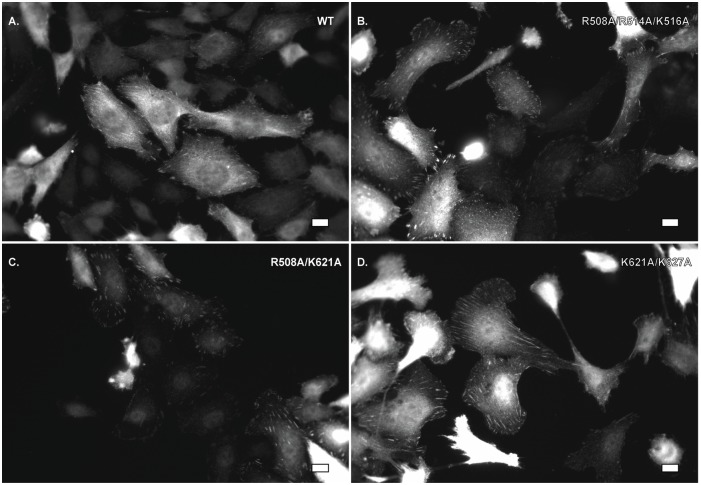
FAK catalytic domain mutants localize to focal adhesions. *fak*-/- MEFs expressing wild type or mutant FAK were plated on fibronectin coated cover slips overnight, fixed, permeabilized and incubated with the anti-FAK 4.47 monoclonal antibody. Alexa Fluor 488-labeled anti-mouse secondary antibody was utilized for visualization. Scale bar = 10 μm.

As localization of FAK is required for correct regulation, the subcellular localization of the FAK mutants was assessed by immunofluorescence to ensure the phosphorylation defects seen were not due to mislocalization. The *fak*^*-/-*^ MEFs exhibited no FAK staining (data not shown), while cells re-expressing wild type or mutant FAK exhibited staining with the anti-FAK antibody. The FAK mutants localized to focal adhesions comparably to wild type (Fig 3). This indicates that the reduced phosphorylation level of the FAK mutants is not attributable to poor localization.

### Catalytic domain mutants exhibit impaired biological function

Knockout of FAK from cells leads to a variety of phenotypes associated with defects in focal adhesion turnover, including an increase in focal adhesion size and decreased cell spreading. To test the biological effectiveness of the catalytic domain mutants, focal adhesion size, focal adhesion dynamics and cell spreading were measured.

**Fig 4:**
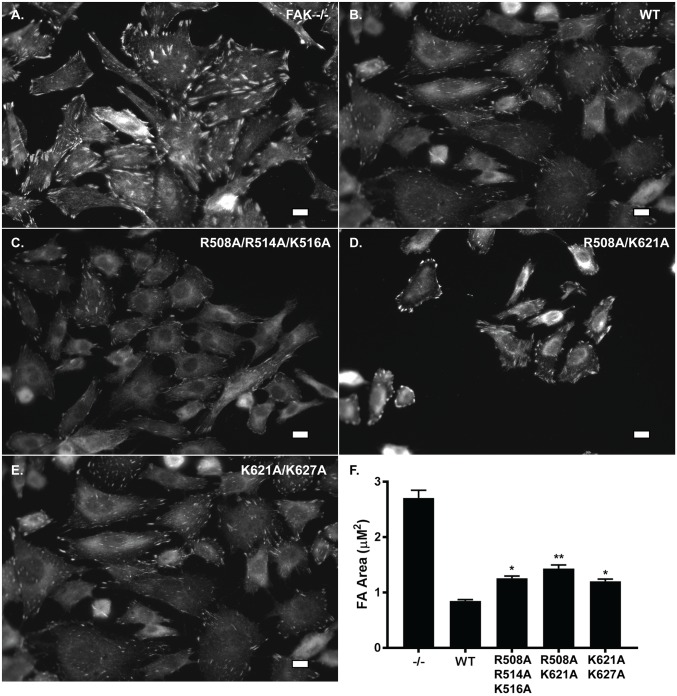
FAK catalytic domain mutants are partially defective in controlling focal adhesion size. **A-E)** *fak*^-/-^ MEFs expressing wild type or mutant FAK were plated on fibronectin coated cover slips, fixed, permeabilized and incubated with an anti-paxillin monoclonal antibody. Alexa Fluor 488-labeled anti-mouse secondary antibody was utilized for visualization. Scale bar = 10 μm. **F**. The average focal adhesion size in each cell line (+/- S.D.) was calculated using ImageJ (*n* > 30 cells for each group, >20 focal adhesions measured per cell, over 3 experiments). Areas were then compared using a one-way ANOVA with a Bonferroni Multiple Comparison post-test. The focal adhesion size in cells expressing each of the FAK proteins was significantly different from the focal adhesion size exhibited by *fak*^*-/-*^ cells (*P* < 0.001). The focal adhesion size in cells expressing the mutant proteins was also significantly different than the focal adhesion size in cells expressing wild type FAK (* *P* < 0.5, ** *P* < 0.01).

*fak*^*-/-*^ MEFs exhibit an increase in focal adhesion size and re-expression of wild type FAK rescues this phenotype [37]. To determine if the catalytic domain mutants were capable of rescuing this phenotype, cells were immunostained for paxillin, a focal adhesion marker, and visualized by immunofluorescence. As previously published, the *fak*^*-/-*^ MEFs exhibited large focal adhesions and cells re-expressing wild type FAK contained much smaller focal adhesions [38] (Fig 4A and 4B). The focal adhesions in cells expressing FAK mutants appeared to have an intermediate focal adhesion phenotype, i.e. paxillin-positive focal adhesions in cells expressing FAK mutants are smaller than those in *fak*^-/-^ cells, but larger than the focal adhesions in wild type FAK-expressing cells (Fig 4C, 4D and 4E). Thus, the mutants appeared partially defective in controlling focal adhesion size.

To confirm this qualitative observation, the average area of the focal adhesions in each of the cells was calculated (Fig 4F). As expected, cells re-expressing wild type FAK had significantly smaller focal adhesions (0.85 ± 0.27 μm) than *fak*^*-/-*^ cells (2.71 ± 0.05 μm). Cells re-expressing the R508A/K621A mutant exhibited significantly larger focal adhesions than cells re-expressing WT FAK (1.43 ± 0.13 μm, *P*<0.01). Cells expressing the other two FAK mutants, R508A/R514A/K515A and K621A/K627A, also exhibited a larger average focal adhesion area than wild type re-expressing cells (1.26 ± 0.08 μm and 1.20 ± 0.08 μm, *P*<0.05) (Fig 4). These size differences demonstrate that these mutants are partially defective for transmitting downstream signals that control a biological outcome regulated by the wild type FAK protein.

The role of FAK function in focal adhesion turnover is well established [39]. To assess the role of catalytic domain residues in regulation of focal adhesion dynamics, focal adhesion stability, assembly and disassembly rates were measured. As R508A/K621A exhibited the largest defect on focal adhesion size, this analysis was focused on this mutant. *fak*^*-/-*^ MEFs stably expressing wild type or R508A/K621A FAK were transfected with GFP-paxillin as a marker to monitor focal adhesion assembly and disassembly. Images were collected at one minute intervals over two hours. Images were analyzed through the Focal Adhesion Analysis Server (FAAS) [33]. Re-expression of wild type FAK in *fak*^*-/-*^ MEFs resulted in an increase in both the rate of assembly and disassembly resulting in a stability time of 18.55 minutes, compared with 30.07 minutes for *fak*^*-/-*^ MEFs (Table 1). Expression of R508A/K621A modestly increased the focal adhesion assembly and disassembly rates and the stability time of focal adhesions in these cells was 25.60 minutes. Thus, these basic residue mutations impair normal FAK function in regulating assembly and disassembly of focal adhesions.

**Table 1:** Summary of results from focal adhesion analysis server.

Cell Type	Assembly Rate (min^-1^)	Disassembly Rate (min^-1^)	Stability Time (min)	n (FA)
WT FAK	1.04 x 10 ^−1^ ± 0.035	1.55x10^-1^ ± 0.017	18.55 ± 0.75	2064
FAK -/-	1.01 x 10^−2^ ± 0.0065	9.88x10^-3^ ± 0.008	30.07 ± 0.35	1285
R508A/K621A	2.02x10^-2^ ± 0.0025	2.97x10^-2^ ± 0.0046	25.60 ± 0.20	1775

**Fig 5:**
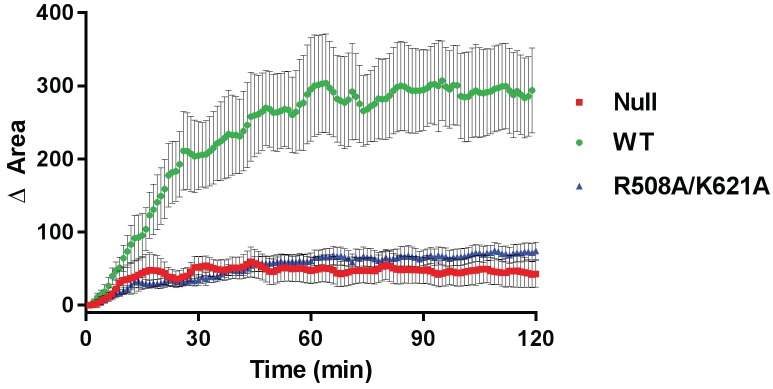
The R508A/K621A mutant is defective at promoting cell spreading. *fak*^-/-^ cells and *fak*^-/-^ cells expressing wild type FAK or the R508A/K621A mutant were transfected with GFP-paxillin as a fluorescent marker for time lapse imaging. Cells were trypsinized, taken into suspension and plated on fibronectin coated imaging dishes. After 5 minute incubation to allow for attachment image acquisition was initiated. Images were captured every minute for 120 minutes and cell diameters were determined using Nikon NIS Elements analysis software. The change in average cell area (+/- S.D.) for each cell type is plotted against time following initiation of imaging. (n = 18–20 cells over 4 experiments)

Since FAK activity is required for rapid cell spreading on fibronectin the ability of the R508A/K621A mutant to promote cell spreading was also assessed [38]. *fak*^*-/-*^ MEFs and cells re-expressing wild type FAK or R508A/K621A were transfected with GFP-paxillin to facilitate imaging. Cells were trypsinized, plated on fibronectin coated coverslips and spreading was monitored by time lapse video microscopy. Images were acquired every minute over a 2 hour period and spreading of individual cells was measured by calculating cell area at each individual time point (Fig 5). While MEFs re-expressing wild type FAK spread rapidly on fibronectin coated coverslips, *fak*^*-/-*^ cells took significantly longer to spread. Cells expressing R508A/K621A exhibited a dramatic defect in cell spreading, comparable to the spreading defect exhibited by the *fak*^*-/-*^ MEFs. These results support the hypothesis that the basic residues on the catalytic domain of FAK are required for biological function.

### FAK catalytic domain binds phospholipids

**Fig 6:**
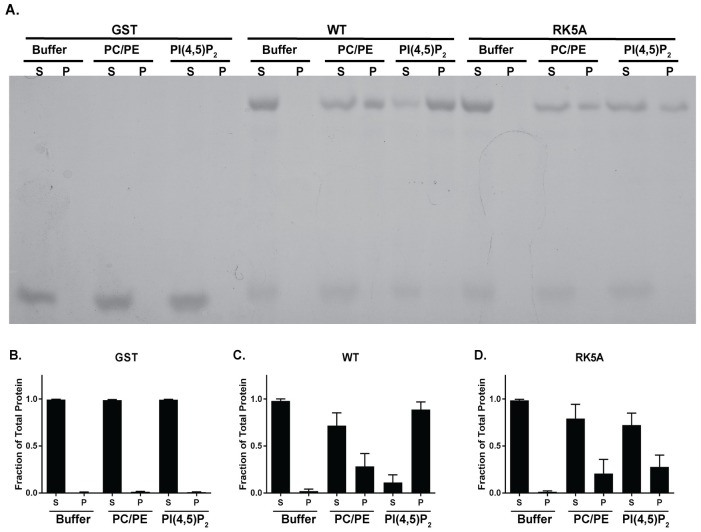
FAK catalytic domain binds PI(4,5)P_2_ containing lipid vesicles. GST fusion proteins were incubated in buffer alone or with large unilamellar vesicles comprised of 60%PC/40%PE (PC/PE) or 50%PC/40%PE/10% PI(4,5)P_2_ (PI(4,5)P_2_). The samples were sedimented at 100,000 x g and the supernatant (S) and pellet (P) fractions analyzed by S.D.S-PAGE and Coomassie blue staining (panel **A**). The results of quantification of multiple experiments (*n* = 6) is shown in panels **B-D**. The partitioning of GST (panel B), the wild catalytic domain of FAK (panel C) and the RK5A mutant catalytic domain (panel D) between supernatant (S) and pellet (P) fractions is shown. Gels were analyzed using ImageJ and the fraction of each protein in the supernatant and pellet (+/- S.D.) was plotted.

Molecular dynamics simulations suggest that five basic residues in the catalytic domain of FAK can dock to PI(4,5)P_2_ head groups in the membrane [28] (Fig 1). To experimentally test this hypothesis, the catalytic domain of FAK was expressed as a GST fusion protein and binding to PI(4,5)P_2_ was measured. Two different experimental approaches were utilized to measure lipid binding, lipid vesicle co-sedimentation and fluorescence anisotropy. For lipid vesicle co-sedimentation studies, large unilamellar lipid vesicles composed of 60% phosphatidylcholine (PC) and 40% phosphatidylethanolamine (PE) were prepared as control vesicles and experimental vesicles containing 10% PI(4,5)P_2_/50%PC/40%PE were made. GST or GST catalytic domain fusion proteins were incubated with lipid vesicles for 1 hour on ice prior to vesicle sedimentation at 100,000 x g for 1 hour at 4°C. The amounts of GST and fusion protein partitioning into the pellet (lipid bound) and supernatant (free) was determined by analyzing the two fractions by S.D.S-PAGE and Coomassie blue staining. In the absence of lipid vesicles, each protein was exclusively found in the supernatant (Fig 6A). In the presence of PC/PE vesicles, a fraction of the GST catalytic domain fusion protein co-sedimented with the vesicles (28.2%). A larger fraction of the GST catalytic domain fusion protein (88.8%) co-sedimented with PI(4,5)P_2_ containing vesicles (Fig 6). Under all conditions, the GST control remained in the supernatant fraction. To determine the role of the catalytic domain basic residues in vesicle binding, a mutant containing alanine substitutions for all 5 of these residues (Arg508, Arg514, Lys515, Lys621 and Lys627) was engineered (called RK5A) and analyzed for co-sedimentation with lipid vesicles. This GST mutant catalytic domain fusion protein was found in the supernatant in the absence of lipid vesicles. In the presence of PC/PE lipid vesicles, a fraction of the mutant fusion protein co-sedimented with the vesicles (20.6%). A similar fraction of the mutant fusion protein co-sedimented with PI(4,5)P_2_-containing vesicles (27.6%) (Fig 6). These findings demonstrate that the FAK catalytic domain exhibits some binding to PC/PE vesicles, that binding is increased in the presence of PI(4,5)P_2_, and that the basic residues on the catalytic domain play a role in binding to PI(4,5)P_2_.

**Fig 7:**
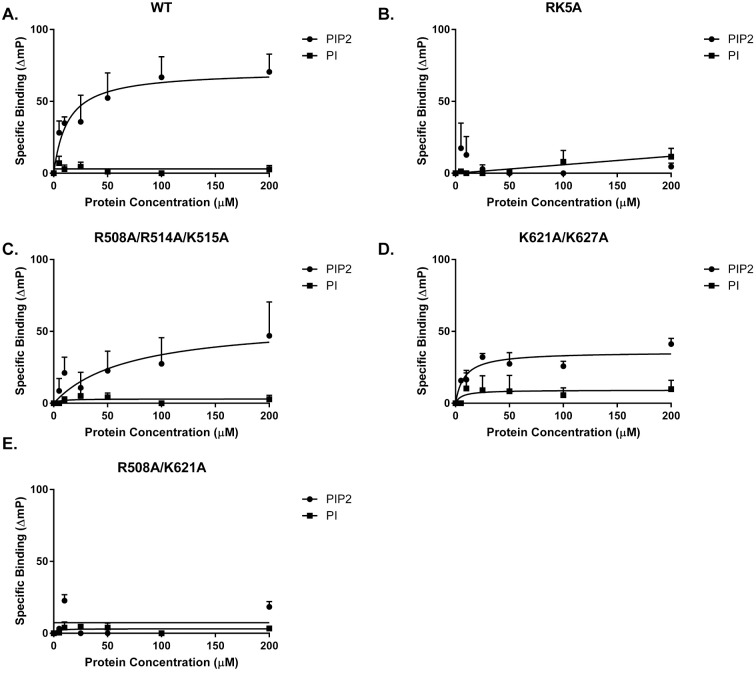
FAK catalytic domain binds short acyl chain PI(4,5)P_2_. FAK catalytic domain GST fusion proteins were incubated with BODIPY labeled short acyl chain (C6) PI or PI(4,5)P_2_ and fluorescence anisotropy measured as the change in mP. Specific binding was determined by subtraction of ΔmP for GST alone from ΔmP for the GST catalytic domain fusion protein. The average change in mP (+/- S.D.) as a function of protein concentration (*n* = 3 experiments) is shown. Specific binding of the wild type catalytic domain (**A**) and fusion proteins containing the RK5A (**B**), R508A/R514A/K515A (**C**), K621A/K627A (**D**) and R508A/K621A (**D**) mutations to PI(4,5)P_2_ was determined. The dissociation constants for the interaction between the catalytic domain variants and PI(4,5)P_2_ was calculated from the specific binding curves. Wild type *K*_*d*_ = 15.7 ± 6.5 μM, RK5A *K*_*d*_ >200 μM, R508A/R514A/K515A *K*_*d*_ = 16.4 ± 3.2 μM, K621A/K627A *K*_*d*_ = 20.8 ± 2.7 μM, R508A/K621A *K*_*d*_ >200 μM.

PI(4,5)P_2_ binding was further validated using fluorescence anisotropy. GST and the GST catalytic domain of FAK were incubated with BODIPY-TMR labeled short acyl chain (C6) PI or PI(4,5)P_2_ and anisotropy measured. Specific binding (ΔmP of GST-catalytic domain minus ΔmP of GST ΔmP of GST) was plotted against protein concentration and Kd for binding was calculated (Kd = 15.7+/-6.5 μM) (Fig 7). The wild type catalytic domain did not specifically bind PI and the RK5A mutant showed no specific binding to PI(4,5)P_2_.

### FAK catalytic domain mutants exhibiting biological defects are defective for phospholipid binding

To determine if the FAK mutants exhibiting defects in the control of biological processes also exhibited defects in phospholipid binding, the R508A/R514A/K515A, K621A/K627A, and R508A/K621A mutations were engineered into the GST-catalytic domain construct. Short acyl chain (C6) BODIPY labeled PI(4,5)P_2_, was chosen to assess the ability of these mutants to bind phospholipids using fluorescence anisotropy. The R508A/R514A/K515A and K621A/K627A mutants each bound to the short acyl chain PI(4,5)P_2_ (*K*_*d*_ = 16.4 ± 3.2 and 20.8 ± 2.7 μM respectively) (Fig 7C and 7D). In contrast, the R508A/K621A mutant was defective for PI(4,5)P_2_ binding (*K*_*d*_ > 200 μM)(Fig 7E). Thus, the mutants exhibiting modest biological defects were capable of binding PI(4,5)P_2_, whereas the mutant with the severest defect in controlling biological responses was deficient in binding PI(4,5)P_2_. These results support a role for these group II basic residues in the phosphatidylinositol phosphate binding activity of the catalytic domain and demonstrate that Arg508 and Lys621 are particularly important for phosphatidylinositol phosphate binding and regulating biological outcomes at the cellular level.

### FAK catalytic domain interacts with multiple phosphatidylinositol phosphates

**Fig 8:**
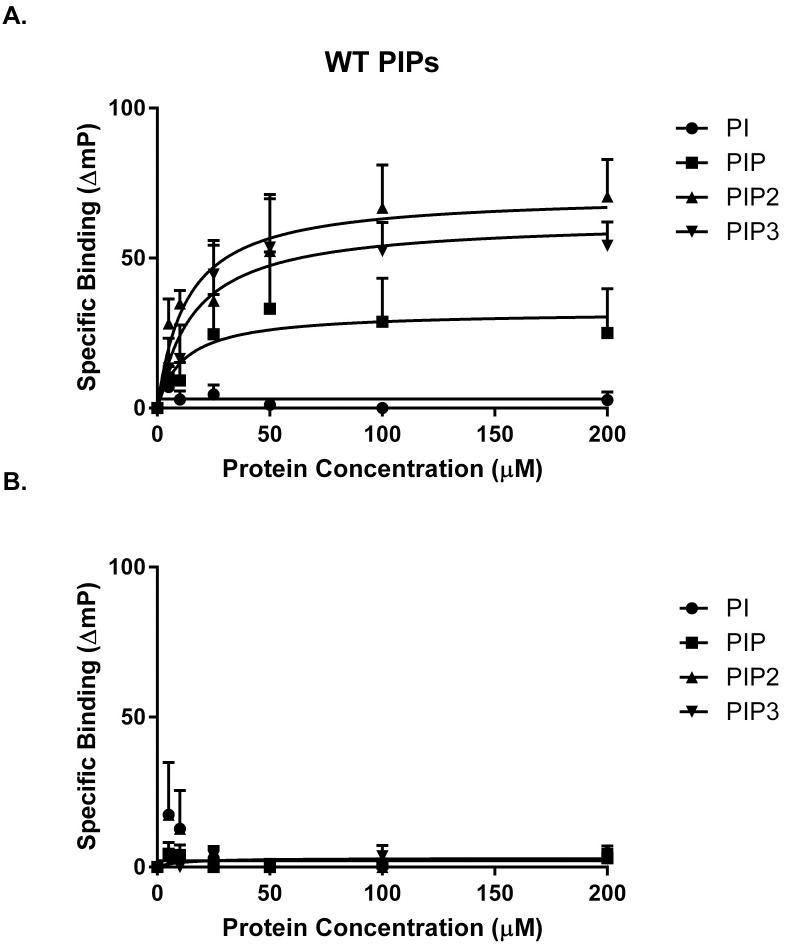
FAK catalytic domain binds multiple phosphatidylinositol phosphates. Fusion proteins were incubated with BODIPY labeled short acyl chain (C6) phospholipids and florescence anisotropy was measured. **A**) ΔmP for BODIPY labeled PI, PI(4)P, PI(4,5)P_2_ and PI(3,4,5)P_3_ in the presence of 100 μM GST, GST wild type catalytic domain and the RK5A mutant is shown (+/-S.D., n = 3 experiments). Specific binding (ΔmP of GST-FAK catalytic domain minus ΔmP of GST alone) of GST wild type catalytic domain (B) and the RK5A mutant (C) is plotted versus concentration. Specific binding to PI (circles), PI(4)P (squares), PI(4,5)P_2_ (triangles) and PI (3,4,5)P_3_ (inverted triangles are shown). The dissociation constant of the wild type catalytic domain for each phosphatidylinositol phosphate was calculated from the curves. PI *K*_*d*_>200 μM, PI(4)P *K*_*d*_ = 11.7 +/- 13.7 μM, PI(4,5)P_2_ *K*_*d*_ = 12.9 +/- 2.3 μM, PI(3,4,5)P_3_ *K*_*d*_ = 16 +/- 9.2 μM.

To test the specificity of the lipid interaction, the binding of wild type and RK5A GST-fusion proteins to phosphatidylinositol, PI(4)P, PI(4,5)P_2_, and PI(3,4,5)P_3_ was analyzed. Purified GST catalytic domain fusion proteins were titrated into a solution of BODIPY-labeled phospholipids with C6-acyl chains, and anisotropy was measured. Phosphatidylinositol with C6-acyl chains and purified GST were used as controls. Differences in anisotropy of each BODIPY-labeled phospholipid at 100 and 200 μM of each protein were compared by ANOVA and a Tukey’s posttest (S1 Table). The wild type catalytic domain showed significant binding to all phosphatidylinositol phosphates, but not PI, compare to the GST control and RK5A. Compared with the PI control, binding of PI(4,5)P_2_ and PI(3,4,5)P_3_ to the wild type catalytic domain was significant, while binding of PI(4)P approached, but did not reach significance. To calculate binding affinities, anisotropy was plotted against protein concentration and binding to GST was subtracted as background binding. The K_d_ of the wild type catalytic domain for PI(4,5)P_2_ was calculated to be 12.9 ± 2.3 μM (Fig 8B). Specific binding of wild type to PI(4)P and PI(3,4,5)P_3_ resulted in K_d_ values of 11.7 ± 13.7 μM and 16 ± 9.2 μM respectively. To determine if the observed interaction with the short acyl chain PI(4,5)P_2_ required the basic residues implicated in binding to PI(4,5)P_2_-containing vesicles, the RK5A mutant was analyzed. This mutant exhibited binding similar to GST with all phospholipids tested (*K*_*d*_>200 μM) (Fig 8C). These results demonstrate that the catalytic domain of FAK exhibits phosphatidylinositol phosphate binding, but that it does not discriminate between different phosphatidylinositol phosphate species.

### Phospholipid binding and catalytic activity

**Fig 9:**
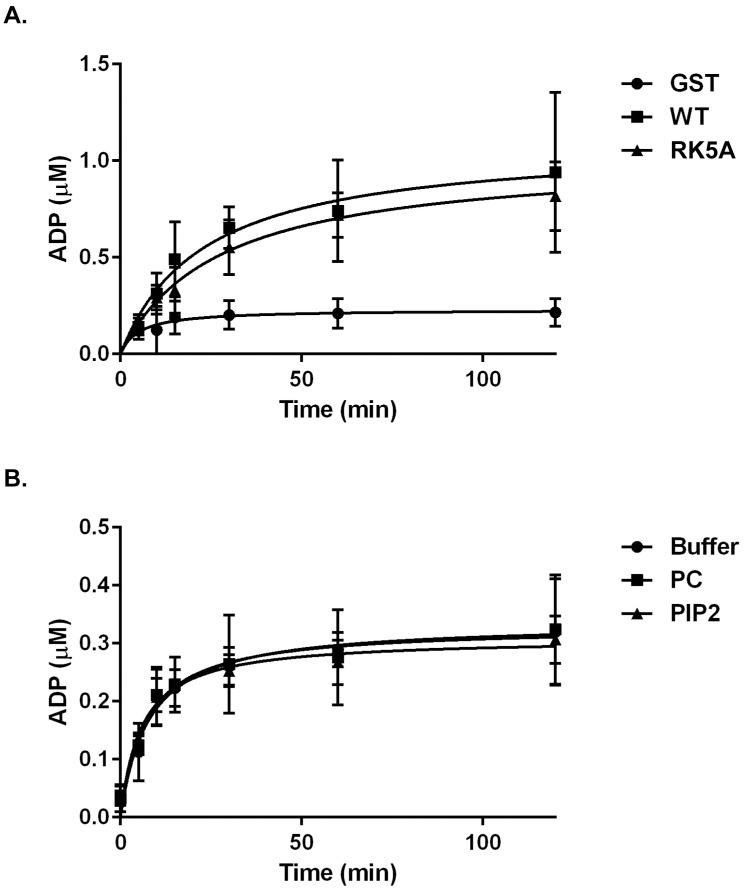
Lipid binding does not modulate activity of isolated FAK catalytic domain. The catalytic activity of GST fusion proteins was determined using poly(Glu, Tyr) as a substrate. The generation of ADP over time was measured using the ADP-Glo^™^ assay protocol. **A**) The kinase activity of GST alone (circles), the GST catalytic domain fusion protein (WT, squares) and the GST RK5A mutant fusion protein (triangles) were measured. The average concentration of ADP (+/- S.D.) generated in each reaction is plotted versus time (*n* = 3). Specific activities were calculated for both WT and RK5A proteins by taking the amount of product produced over a 5 minute period of time during the initial, linear portion of the reaction divided by time, divided by mass of fusion protein. This resulted in a specific activity of 1.025 +/- 0.002 nmol/min/mg for GST-WT and 0.913 +/- 0.004 nmol/min/mg for GST-RK5A. **B**) WT catalytic domain was pre-incubated in lipid sedimentation buffer (squares), buffer containing 60%PC/40%PE vesicles (triangles), or buffer containing 50%PC/40%PE/10%PI(4,5)P_2_ vesicles (circles) for one hour prior to performing the kinase assay. The average ADP concentration (+/- S.D.) generated in each reaction is plotted versus time (*n* = 3) Specific activities were calculated for all conditions by taking the amount of product produced over a 5 minute period of time during the initial, linear portion of the reaction divided by time, divided by mass of fusion protein. This resulted in a specific activity of 0.460 +/- 0.004 nmol/min/mg for buffer containing 60%PC/40%PE vesicles and 0.543 +/- 0.006 nmol/min/mg for buffer containing 50%PC/40%PE/10%PI(4,5)P_2_ vesicles.

Although it was unlikely that substitution of basic residues on the surface of the catalytic domain with alanine residues would perturb enzymatic activity, catalytic activity of the wild type and RK5A mutant fusion proteins was measured. Four micrograms of protein were incubated in kinase reaction buffer containing poly(Glu,Tyr) as substrate and the generation of ADP was monitored using the ADP-Glo assay. GST alone showed no activity while the wild type GST catalytic domain demonstrated activity. The catalytic activity of the RK5A mutant was identical to the activity of the wild type fusion protein (Fig 9A). Therefore the mutations did not alter the enzyme activity of the catalytic domain and by inference did not perturb the structure of the domain.

An intriguing possibility is that lipid binding to the catalytic domain might directly modulate catalytic activity. To test this hypothesis, the GST catalytic domain fusion protein was incubated with PC/PE vesicles or PC/PE vesicles containing PI(4,5)P_2_ in vesicle binding buffer, prior to performing the kinase assay. The catalytic domain exhibited the same activity in the presence of buffer only, PC/PE vesicles and PC/PE vesicles containing PI(4,5)P_2_ (Fig 9B). These results demonstrate that association with lipid vesicles has no impact upon the enzymatic activity of the isolated catalytic domain.

## Discussion

In addition to studies demonstrating PI(4,5)P_2_ binding to the basic sequence on the F2 lobe of the FERM domain [24,25], a recent computational modeling study identified other potential PI(4,5)P_2_ interaction sites on the catalytic domain [28]. We have validated PI(4,5)P_2_ binding to the catalytic domain using both lipid sedimentation and fluorescence anisotropy approaches and mutation of the basic residues proposed to bind PI(4,5)P_2_ impaired catalytic domain binding to PI(4,5)P_2_. While identified basic residues did mediate this interaction, the catalytic domain of FAK also demonstrated binding to PI(4)P and PI(3,4,5)P_3_. Thus the basic residues on the surface of the catalytic domain facilitate association with multiple phosphatidylinositol phosphates rather than a specific phosphorylated species.

Interestingly, mutation of a pair of spatially proximal basic residues, R508A/K621A, was sufficient to dramatically impair phosphatidylinositol phosphate binding. This mutant exhibited defects in tyrosine phosphorylation *in vivo* and in the control of biological events regulated by FAK. These results implicate these catalytic domain basic residues in FAK function *in vivo*, which can at least partially be attributed to defects in phospholipid binding. Mutation of other subsets of the catalytic domain basic residues had little impact on phosphatidylinositol phosphate binding in the fluorescence anisotropy assay and only partially impaired the control of FAK-dependent biological responses in cells. The R508A/R514A/K515A and K621A/K627A mutants could exhibit a more subtle phospholipid binding defect, not detected in the fluorescence anisotropy experiments, or a defect in association with a novel protein or ligand. All five of these residues are conserved, in avian, murine and human FAK sequences. R508, K621 (the most important residues for biological function) and K627 are also conserved in *Drosophila melanogaster*. Interestingly, only one of these residues (K515) is conserved in the murine and human sequences of protein tyrosine kinase 2 (PYK2), a protein highly related in sequence to FAK [40]. In total, these observations support the importance of these basic residues in the biochemical and biological function of FAK and suggest a function that is unique to FAK and not shared by Pyk2 [40] [41,42].

Phosphatidylinositol phosphate binding is a key component in many cellular signaling pathways and can serve to alter subcellular localization and/or enzymatic activity. A very well characterized example is the PI(3,4,5)P_3_/Akt signal transduction pathway, where the generation of PI(3,4,5)P_3_ at the membrane recruits protein kinase D and Akt to the membrane via their PH domains facilitating activation of Akt [43]. The mechanism of PH domain binding to phospholipids is well established. PH domains accommodate the headgroup of phosphatidylinositol phosphates, for example PI(3,4,5)P_3_ and PI(4,5)P_2_, in a basic pocket that recognizes charge and shape to provide phospholipid binding specificity [44,45] A number of other lipid binding domains, e.g. FYVE and ENTH domains, similarly use a basic pocket to bind phosphoinositides [46,47]. In contrast, there are specific examples of phosphatidylinositol phosphate binding to basic residues exposed on the surface of a domain, rather than within a binding pocket. Examples of this binding mechanism include the FERM domain of FAK, which binds PI(4,5)P_2_ through interactions with basic residues along a surface exposed α-helix [22], and the tail of vinculin, which interacts with PI(4,5)P_2_ through interactions with basic residue side chains projecting from its surface [48]. Other modular domains exhibit phospholipid binding sites that do not contain a basic binding pocket. For example many phosphotyrosine binding (PTB) domains have phospholipid binding sites separate from their phosphotyrosine binding sites. The phospholipid binding motif in PTBs is not a conserved sequence but an electrostatic feature defined as a “basic crown” [49], and mutation of basic residues in these regions can abrogate lipid binding [50]. Additionally, a recent study showed that many Src homology 2 (SH2) domains bind plasma membrane lipids with a high affinity and that binding occurs through alternate cationic patches (ACPs) [51]. ACPs bind several membrane lipid molecules simultaneously while leaving the phosphotyrosine binding site of the SH2 domain accessible for ligand binding.

The interaction of the FAK catalytic domain with the membrane via basic residues on the side of the catalytic domain is envisioned to leave the ATP and substrate binding sites accessible for catalytic activity. A few studies have address the structure of substrates in complex with kinase domains using peptides to mimic the interaction with the active site. In these structures, the substrates adopt an extended conformation [52–55]. Further, computational analysis reveals that phosphorylation sites reside in regions of proteins that are predicted to be disordered [56]. A main phosphorylation target of FAK catalytic activity is an autophosphorylation site, tyrosine 397 in the flexible linker between the FERM and catalytic domains [57]. In the autoinhibitied conformation, the linker region containing tyrosine 397 binds to the FERM domain. Biochemical analysis reveals that in this conformation tyrosine 397 is a poor site for phosphorylation, compared with an extended conformation, suggesting that release of the linker from the FERM domain may be required for phosphorylation (REF—Lietha 2007). There is no structural information about the linker in any context other than the autoinhibited conformation, and predictions suggest that it is unstructured. A second substrate is the FAK binding protein, paxillin. The N-terminal half of paxillin, which is the location of its phosphorylation sites, is predicted to be intrinsically disordered. Since authentic FAK substrates are phosphorylated in disordered regions, we expect that these sites can access the FAK active site, even when adjacent to the membrane.

The phosphatidylinositol phosphate binding site on the catalytic domain, which lies on the side of the catalytic domain near the juncture of the small and large lobes of the kinase is at a site that could potentially regulate catalytic activity. The interaction of other ligands with the catalytic domain of kinases can alter activity. For example, binding of cyclin to cyclin-dependent kinases alters the small lobe of the catalytic domain to create a catalytically competent structure [58]. Small ligands can also impact activity. For example, copper binding to the catalytic domain of MEK1 is required for enzymatic activity [59]. While these ligands bind on the opposite side of the kinase domain relative to the phosphatidylinositol phosphate binding site on the FAK catalytic domain, these precedents prompted an experiment to directly assess the impact of lipid binding upon catalytic activity. The results demonstrated that lipid binding has no effect upon the activity of the isolated FAK domain. These results are consistent with the conclusion drawn by Goni et al, that phosphatidylinositol phosphate binding does not directly regulate catalytic activity [25]. Since lipid binding does not directly regulate the activity of the isolated catalytic domain, the observed biochemical and biological defects associated with the phosphatidylinositol phosphate binding mutant reflects a role in regulating function only in the context of the full length FAK protein.

**Fig 10:**
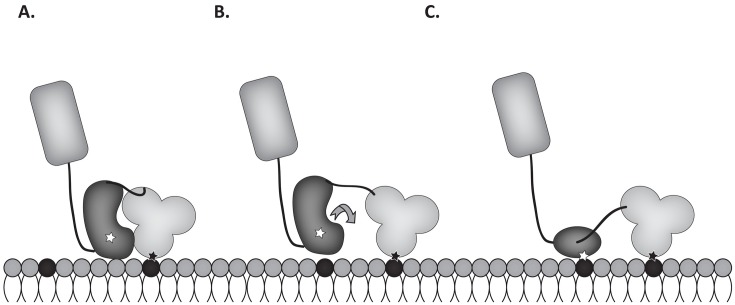
Proposed model of FAK activation. **A)** FAK in its autoinhibited conformation can dock to PI(4,5)P_2_ (black) via the basic sequences in the FERM domain (black star in light gray domain). The interaction between the FERM and catalytic domains (dark gray) is disrupted via a poorly defined mechanism (**B**), allowing the rotation of the catalytic domain and docking to membrane phospholipids via the basic sequences in the catalytic domain (white star) (**C**).

Goni, et al. report lipid binding experiments with a construct containing the FERM and catalytic domains of FAK, and the KAKTLRK mutant, which ablates the FERM domain basic patch, does not exhibit binding despite the presence of the basic residues in the catalytic domain [25]. The difference in results between studies could reflect differences in experimental conditions including vesicle and buffer composition. Conversely, the different results might demonstrate that PI(4,5)P_2_ engagement of the FERM domain basic patch is a requirement for catalytic domain binding to phospholipids in the context of a larger construct, although such a requirement was not evident in molecular dynamics simulations. Interestingly, phospholipid binding studies using the construct containing the FERM domain and catalytic domain demonstrated cooperative binding [25]. This could reflect oligomerization of FAK [25]. However, the cooperativity of binding could also be explained by exposure of a second phosphatidylinositol phosphate binding site, i.e. the binding site in the catalytic domain. The critical mechanistic event relieving autoinhibition resulting in FAK activation remains to be conclusively demonstrated. PI(4,5)P_2_ binding to the FERM domain is required, but insufficient for FAK activation [25]. It is possible that upon FERM domain binding dissociation of the FERM and catalytic domains must occur prior to docking of the catalytic domain to phosphatidylinositol phosphates. It has not been conclusively established how the FERM domain/catalytic domain interface interactions are disrupted during FAK activation. An attractive model for activation is a mechanical mechanism, since FAK activation is sensitive to stiffness of the extracellular matrix outside the cell and requires the integrity of the cytoskeleton inside the cell [60,61]. A recent molecular dynamics simulation has demonstrated that sufficient force can disrupt the interaction between the FERM and catalytic domains without dissociating the FERM domain from the PI(4,5)P_2_ and without disrupting the alpha helical structure of the FERM and catalytic domains [27]. Given this consideration, the catalytic domain interaction with phospholipids in the membrane may serve to stabilize FAK in an active conformation, rather than participate in the activation mechanism (Fig 10).

## Supporting information

S1 Table Statistical analysis of binding. Differences in anisotropy of each BODIPY-labeled phospholipid at 100 and 200 μM of each protein were compared by ANOVA and a Tukey’s posttest, p values for each comparison are shown.
(DOCX)

## Abbreviations

ACP: alternate cationic patches
ADP: adenosine diphosphate
ATP: adenosine triphosphate
BODIPY: dipyrromethene boron difluoride
FAK: Focal Adhesion Kinase
FERM: band 4.1, Ezrin, Radixin, Moesin domain
GFP: green fluorescent protein
GST: glutathione-S-transferase
LMV: large multilamellar vesicle
LUV: large unilamellar vesicle
PC: phosphatidylcholine
PE: phosphatidylethanolamine
PH: pleckstrin homology
PI(4,5)P_2_: Phosphatidylinositol-4,5-bisphosphate
PTB: phosphotyrosine binding
SH2: Src homology 2
