# Supplementary material for: Phospholipid binding to the FAK catalytic domain impacts function
Source: PLoS One. 2017 Feb 21;12(2):e0172136. doi: 10.1371/journal.pone.0172136 (PMC5319746; doi:10.1371/journal.pone.0172136)
Supplement: S1 Table — Differences in anisotropy of each BODIPY-labeled phospholipid at 100 and 200 μM of each protein were compared by ANOVA and a Tukey’s posttest, p values for each comparison are shown. (DOCX) [file pone.0172136.s001.docx]

**Supporting Table 1. Statistical analysis of binding**. Results of ANOVA and Tukey’s post test

| **Comparison** | **Protein Concentration** | **p value** |
| --- | --- | --- |
| **PI** vs **PI(4)P** binding to catalytic domain | 100 µM | 0.069 |
| **PI** vs **PI(4,5)P_2_** binding to catalytic domain | 100 µM | 0.002 |
| **PI** vs **PI(3,4,5)P_3_** binding to catalytic domain | 100 µM | 0.030 |
| **PI** vs **PI(4)P** binding to catalytic domain | 200 µM | 0.058 |
| **PI** vs **PI(4,5)P_2_** binding to catalytic domain | 200 µM | 0.048 |
| **PI** vs **PI(3,4,5)P_3_** binding to catalytic domain | 200 µM | 0.005 |
| **PI** binding to **catalytic domain** vs **GST** | 100 µM | 1.000 |
| **PI(4)P** binding to **catalytic domain** vs **GST** | 100 µM | 0.050 |
| **PI(4,5)P_2_** binding to **catalytic domain** vs **GST** | 100 µM | 0.015 |
| **PI(3,4,5)P_2_** binding to **catalytic domain** vs **GST** | 100 µM | 0.029 |
| **PI** binding to **catalytic domain** vs **GST** | 200 µM | 1.000 |
| **PI(4)P** binding to **catalytic domain** vs **GST** | 200 µM | 0.038 |
| **PI(4,5)P_2_** binding to **catalytic domain** vs **GST** | 200 µM | 0.010 |
| **PI(3,4,5)P_2_** binding to **catalytic domain** vs **GST** | 200 µM | 0.028 |
| **PI** binding to **WT** vs **RK5A** catalytic domain | 100 µM | 1.000 |
| **PI(4)P** binding to **WT** vs **RK5A** catalytic domain | 100 µM | 0.064 |
| **PI(4,5)P_2_** binding to **WT** vs **RK5A** catalytic domain | 100 µM | 0.044 |
| **PI(3,4,5)P_2_** binding to **WT** vs **RK5A** catalytic domain | 100 µM | 0.054 |
| **PI** binding to **WT** vs **RK5A** catalytic domain | 200 µM | 1.000 |
| **PI(4)P** binding to **WT** vs **RK5A** catalytic domain | 200 µM | 0.031 |
| **PI(4,5)P_2_** binding to **WT** vs **RK5A** catalytic domain | 200 µM | 0.038 |
| **PI(3,4,5)P_2_** binding to **WT** vs **RK5A** catalytic domain | 200 µM | 0.043 |
